# Acclimation to high temperature during pollen development

**DOI:** 10.1007/s00497-016-0282-x

**Published:** 2016-04-11

**Authors:** Florian Müller, Ivo Rieu

**Affiliations:** Department of Molecular Plant Physiology, Institute for Water and Wetland Research, Radboud University, Nijmegen, The Netherlands

**Keywords:** High temperature response, Heat stress, Pollen development, Male fertility, Acclimation, Tapetum

## Abstract

*****Key message***:**

**Pollen heat acclimation.**

**Abstract:**

As a consequence of global warming, plants have to face more severe and more frequently occurring periods of high temperature stress. While this affects the whole plant, development of the male gametophyte, the pollen, seems to be the most sensitive process. Given the great importance of functioning pollen for the plant life cycle and for agricultural production, it is necessary to understand this sensitivity. While changes in temperature affect different components of all cells and require a cellular response and acclimation, high temperature effects and responses in developing pollen are distinct from vegetative tissues at several points. This could be related to specific physiological characteristics of developing pollen and supporting tissues which make them vulnerable to high temperature, or its derived effects such as ROS accumulation and carbohydrate starvation. But also expression of heat stress-responsive genes shows unique patterns in developing pollen when compared to vegetative tissues that might explain the failure to withstand high temperatures. As an alternative to viewing pollen failure under high temperature as a result of inherent sensitivity of a specific developmental process, we end by discussing whether it might actually be an adaptation.

## Introduction

Plants are exposed to an ever-changing biotic and abiotic environment and need to constantly adapt their development and physiology to maintain organismal and cellular homeostasis (also referred to as acclimation). An environmental parameter that is highly variable, over various time scales, is ambient temperature. High temperatures reached during the day can pose various problems for cellular functioning and strongly affect plant fitness in the longer term. As a consequence of global warming, hot days and heat waves are predicted to increase both in frequency and in intensity in many temperate regions in the coming decades (IPCC 2014). Given the almost complete dependency of humans on agricultural output for food, understanding the reaction of plants to high temperature stress is of great societal importance. While the majority of studies on this subject have focussed on the vegetative (sporophytic) stage of plant growth, the development and functioning of the male gametophyte, or pollen, are known to be among the most temperature-sensitive processes within the plant life cycle (Zinn et al. 2010). Importantly, heat-induced pollen defect is associated with reductions in seed and fruit set. In this review, we will specifically discuss the high temperature sensitivity and acclimation response of developing pollen and see how this compares to that of vegetative tissues. We will also speculate whether previous experience of high temperature by a plant may induce higher tolerance of pollen towards subsequent temperature increases, i.e. leads to acquired thermotolerance, either within an individual or in the offspring, and discuss ways in which pollen thermotolerance may be enhanced.

## The effect of heat on pollen

### Pollen development

Pollen, the mature male gametophyte (microgametophyte), is a highly specialized cell type that develops within the anthers of the flower through a complex series of processes. This has been reviewed extensively (Borg et al. 2009). During anther development, the reproductive or sporogenous cells, located centrally within the anther, give rise to the pollen mother cells (PMCs; microsporocytes), while the surrounding non-reproductive cells form sporophytic epidermal, cortical and tapetal cell layers. Pollen development from PMCs can be divided into microsporogenesis and microgametogenesis. During microsporogenesis, PMCs undergo a meiotic division, with the four haploid products (spores) of each meiocyte initially staying together in the form of a tetrad. These tetrads are enclosed by a thick wall, mainly consisting of callose, and surrounded by the locular fluid inside the anther locules. The innermost cell layer of the locule differentiates as the tapetum, a tissue that is essential for microsporogenesis through secreting nutrients, carbohydrates, cell wall components and enzymes into the locular fluid. Among these are callases that digest the callose walls of the tetrads, which then release the unicellular microspores. During subsequent microgametogenesis, the microspores undergo vacuolization, expansion and a mitotic, asymmetric division, resulting in the formation of binuclear pollen grains, harbouring a larger vegetative and smaller generative cell. At this stage, the tapetum undergoes programmed cell death. Pollen will then maturate and desiccate. In the case of tri-nucleate pollen, a second mitotic division of the generative cell into two sperm cells occurs before desiccation, while in binucelate pollen grains this happens after pollen germination.

### Heat-induced pollen defects

Developing microspores and pollen have been known for a long time to be the cells that are most affected by the occurrence of high ambient temperatures (Iwahori 1965). Both short-term high and long-term mildly elevated day and night temperatures negatively affect pollen development. An important question is what the primary heat-induced developmental defect(s) during pollen development are and how this differs between heat profiles.

The earliest heat-induced developmental defects occur during meiosis. Next to increased frequency of crossing over and homologous recombination (Boyko et al. 2005; Francis et al. 2007; Lebel et al. 1993), chromosome behaviour and meiotic cell division may be affected, leading to unbalanced chromosome separation between spores and formation of diploid dyads (Omidi et al. 2014; Pecrix et al. 2011; Rezaei et al. 2010). In the study of Pecrix et al. (2011), closer investigation revealed that the behaviour of chromosomes during cell division was due to aberrant spindle orientation. High temperatures are known to affect microtubules and cytoskeleton dynamics, which has been studied in vegetative tissues, as well as during pollen tube growth (Muller et al. 2007; Parrotta et al. 2016; Smertenko et al. 1997). While this requires temperatures of above 40 °C in vegetative cells in Arabidopsis or tobacco, growing pollen tubes are more sensitive (35 °C, 3 h) and damage increases with increasing temperature (Parrotta et al. 2016). In agreement with this, aberrant behaviour of chromosomes during meiosis seems to occur especially under more severe heat stress (De Storme and Geelen 2014).

Defects in microsporogenesis have been described in a number of species, both under extreme heat and long-term mild heat profiles (Ahmed et al. 1992; Endo et al. 2009; Kim et al. 2001; Sato et al. 2002). It has been suggested that a reduction in pollen number and viability might be the indirect result of defects in the supportive tapetal cells (De Storme and Geelen 2014; Parish et al. 2012). Aberrations in the timing of tapetum development and degeneration, including hypertrophy and premature as well as delayed degeneration, and morphology of tapetal endoplasmic reticulum have been observed (Abiko et al. 2005; Ahmed et al. 1992; Endo et al. 2009; Harsant et al. 2013; Iwahori 1965; Oshino et al. 2007; Saini et al. 1984; Suzuki et al. 2001). Similar tapetal defects are known from cold and drought stress and occur in different plant species, both monocots like wheat, barley, Brachypodium distachyon and rice, and dicots like cowpea (Vigna unguiculata), snap bean (Phaseolus vulgaris), Arabidopsis and tomato, always associated with reduced pollen viability (De Storme and Geelen 2014; Parish et al. 2012).

Finally, the amount of starch and sugars in maturing pollen grains has been shown to be affected by long-term, mildly elevated temperature. In pollen of bell pepper and tomato, starch has been shown to accumulate during development and reach a maximum after the first pollen mitosis, a few days before flower anthesis. Subsequently, the starch content is reduced again and the concentration of soluble sugars increases (Aloni et al. 2001; Pressman et al. 2002). When grown under continuous mild heat (32 °C/26 °C day/night), the transient accumulation of starch, as well as the final accumulation of soluble sugars, was reduced in developing tomato pollen, correlating with reduced pollen viability, and tomato cultivars with higher pollen thermotolerance were able to maintain higher starch and sugar levels than heat-sensitive lines (Firon et al. 2006; Pressman et al. 2002). It could be speculated that high temperatures lead to depletion of these reserves due to increased respiration to sustain adaptive metabolic activity in developing pollen. Alternatively, the carbohydrate-related defects may be the result of decreased hexose supply by the tapetum or due to developmental aberrations during earlier microsporogenesis.

How heat affects the above developmental processes remains to be determined. Studies in vegetative cells have identified several cellular effects of high temperature, including increased membrane fluidity, misfolding of proteins, changes in the specificity and kinetics of enzyme reactions and accumulation of reactive oxygen species (ROS) (Alfonso et al. 2001; Atkin and Tjoelker 2003; Sangwan et al. 2002).

## Heat responses during pollen development

To cope with the various effects of high temperatures and to maintain cellular homeostasis, plants have a sophisticated heat stress response. While this is well studied in vegetative stages of different plant species, especially using Arabidopsis seedlings and tomato cell cultures (Kotak et al. 2007; Scharf et al. 2012), little is known about these mechanisms in developing pollen. Transcriptomic studies of developing Arabidopsis and maize (*Zea mays*) pollen have shown that, in comparison with vegetative and other generative tissues, developing pollen is a relatively unique cell type (Becker et al. 2003; Davidson et al. 2011). This means that knowledge obtained from vegetative stages is not necessarily applicable to developing pollen and argues for performance of pollen-specific studies.

### Heat sensing

Heat stress leads to broad transcriptomic changes in plants. Genes differentially expressed under high temperatures include heat stress transcription factors (HSFs) and heat shock proteins (HSPs), but they only a account for small proportion of total transcriptomic changes. Transcriptomic studies in wheat and Arabidopsis showed that 5–10 % of all transcripts were differentially expressed under short heat stress, including genes that putatively encode proteins and transcription factors involved in phosphorylation, hormone biosynthesis and signalling, calcium, sugar and lipid signalling pathways, regulation of transcription and translation, primary and secondary metabolism and responses to different biotic and abiotic stresses (Larkindale and Vierling 2008; Mittler et al. 2012; Qin et al. 2008). Prior to these transcriptomic adjustments, plants have to sense changes in temperatures. Four systems have been described that can sense temperature changes and are thought integrate these to induce heat-responsive gene expression (Mittler et al. 2012). An increase in membrane fluidity is among the first consequences of increasing temperatures, and a calcium channel located in the plasma membrane is considered to be the main sensor. Activated by increasing temperatures, it leads to the accumulation of Ca^2+^ in the cytoplasm and the expression of heat-induced genes, for example by feeding into the HSF pathway as discussed later (Balogh et al. 2013; Mittler et al. 2012). Second, proteins that unfold due to increasing temperatures are sensed by the cytoplasmic and ER unfolded protein response (UPR) and serve as a thermosensor (Moreno and Orellana 2011). The UPR in the cytoplasm involves HSFA2, a major regulator of the HSR, and certain splice variants of HSFA2. In the endoplasmic reticulum, the presence of unfolded proteins leads to the release of bZip transcription factors that enter the nucleus and lead to the expression of heat stress-responsive genes as well (Che et al. 2010). Thirdly, the early accumulation of reactive oxygen species (ROS) is considered one of the first steps in the heat stress signalling cascades. While ROS are constantly produced under normal conditions, especially in mitochondria, chloroplasts and peroxisomes, and directly detoxified by different pathways within these organelles or their vicinity, under high temperatures the balance between production and detoxification seems to be disturbed, leading to the accumulation of ROS (Sharma et al. 2012). And last, a specific histone variant, H2A.Z that is incorporated into nucleosomes especially around the transcriptional start site of genes, seems to regulate nucleosome occupancy at this position in a temperature-sensitive manner (Kumar and Wigge 2010). In a model proposed by Kumar and Wigge (2010), the occupancy of H2A.Z containing nucleosomes declines with increasing temperatures allowing the progression of the polymerase II and transcriptional regulators to access gene-specific regulatory cis-elements, normally occluded by nucleosomes.

It seems likely that the same systems act in developing pollen. Indeed, recently, a calcium channel has been determined to be important for thermotolerance (cycling between 40 °C day and −1 °C night temperature) of Arabidopsis pollen (Tunc-Ozdemir et al. 2013). Mutant plants showed no defects under control conditions, but were more sensitive to high temperature stress and failed to induce expression of heat-responsive transcription factors. Also, accumulation of ROS in pollen upon a short heat shock (42 °C, 2 h) has been reported (Kumar et al. 2014), and this may play a role in acclimation.

### Heat stress transcription factors

Central to the HSR is a network of heat shock transcription factors (HSF) that can bind a specific palindromic DNA sequence, the heat shock element (HSE), and induce the expression of heat-responsive genes (for review Kotak et al. 2007; Scharf et al. 2012). Tomato, for example, possesses 27 different HSFs that can be divided into three different clades (Scharf et al. 2012). These contain different motifs, among which a DNA-binding domain to recognize HSE and an oligomerization domain to form heterooligomers. The division into three different clades is based on structural differences in this oligomerization domain. Under normal temperature conditions, HSFA1, the master regulator of the HSR is located in the cytoplasm and kept inactive by interaction with HSP70 and HSP90 (Hahn et al. 2011). Upon high temperature stress, HSFA1 is activated and together with HSFB1, another HSF that rapidly accumulates under HT and acts as co-activator induces the expression of heat-responsive genes that help to maintain cellular homeostasis and different HSFs that further amplify the HSR (Liu et al. 2011). Among these heat-induced HSFs is HSFA2, which, by oligomerization with HSFA1, forms a so-called superactivator complex that amplifies the HSR (Chan-Schaminet et al. 2009).

High temperature-induced expression of different HSFs has been reported in developing pollen of different species. HSFA2 and HSFA3 are upregulated in tomato microspores, and HSFA2 and HSFB1 are also upregulated in developing pollen of Arabidopsis under a short heat shock (Frank et al. 2009; Giorno et al. 2010; Tunc-Ozdemir et al. 2013). Similar to what was found for vegetative tissue, HSFA2 suppression reduced the tolerance of tomato pollen towards a short high temperature stress (39 °C, 3 h) during the stages of meiosis and early microspore formation (Fragkostefanakis et al. 2016). On the other hand, HSF function also seems to diverge between leaf and anther to some extent, because a considerable difference in the heat-induced genes was found between leaf and anther, as well as in the set of HSFA2-dependent genes (Fragkostefanakis et al. 2016). And, while in vegetative stages of tomato, HSFA2 is solely expressed under high temperature conditions, HSFA2 transcripts are already abundant under control conditions in young developmental stages of tomato pollen (Fragkostefanakis et al. 2016; Frank et al. 2009; Giorno et al. 2010); thus, HSFs might play an additional, developmental role in pollen development. The same expression pattern is found for AtRen1, an HSFA5-like gene that is important for pollen development. Knockout of Ren1 leads to abnormal pollen development, as well as to higher temperature sensitivity of developing pollen (Renak et al. 2014). Since nucleolar appearance was different, the authors concluded that Ren1 might be related to RNA biogenesis. HSFA5, the closest homologue of Ren1, is also upregulated in developing pollen of Arabidopsis and soy bean, but at later stages than Ren1 (Haerizadeh et al. 2009; Renak et al. 2014). In a tomato protoplast system, HSFA5 was found to inhibit the function of HSFA4, a positive regulator of the HSR also expressed in developing pollen (von Koskull-Doring et al. 2007). Taken together, from the few HSFs that have been studied in pollen under high temperatures, it seems that most of them are induced in response to high temperature like in vegetative tissues, suggesting that HSFs play a similar, major role in the heat response in pollen. Additional functional genomic studies are needed to test this hypothesis.

### Heat shock proteins

Protecting and stabilizing proteins in their native conformation are one of the most important aspects for cells to survive high temperatures stress. This is carried out by high-molecular-weight chaperones called heat shock proteins (HSPs). Especially under high temperature, cells accumulate massive amounts of these proteins to prevent irreversible high temperature damage (Vierling 1991). HSPs are divided into classes according to their molecular weight in kDa (HSP100, HSP90, HSP70, HSP60, HSP40 and small HSP with low molecular weights) and stabilize unfolding proteins, prevent the formation of aggregates, resolubilize aggregated proteins and return them to their native conformation (Hartl et al. 2011; Kotak et al. 2007; Vierling 1991). Low-molecular-weight HSPs also play roles in maintaining the cell membrane integrity (Tsvetkova et al. 2002).

In young developmental stages of tomato pollen, several small HSPs and HSP70 are abundant, suggesting a developmentally controlled process that might help to prepare the cells for environmental stresses (“developmental stress priming”; Chaturvedi et al. (2013); Gagliardi et al. (1995); Volkov et al. (2005)). This fits with the finding that HSFA2 is expressed and activates some of its targets in young anthers under non-stress conditions (Fragkostefanakis et al. 2016; Giorno et al. 2010). Furthermore, HSPs and small HSPs are induced in pollen after a short intense high temperature stress (Chaturvedi et al. 2015; Frank et al. 2009; Kumar et al. 2014). However, this might not apply to all HSPs typically expressed in vegetative stages: studies in different species have shown that in developing, mature and germinating pollen, certain heat-responsive proteins, like HSP100, HSP70 and small HSPs, accumulate less under high temperatures than in vegetative tissue (Cooper et al. 1984; Dupuis and Dumas 1990; Gagliardi et al. 1995; Volkov et al. 2005). A recent study in tomato comes to a similar conclusion, namely that HSFA2, HSP100 and HSP17 are upregulated in developing pollen in response to a short heat stress (39 °C, 3 h), but to a considerably lesser extent than in vegetative tissues (Fragkostefanakis et al. 2016). Similarly, promotor-GUS fusions have shown that in response to high temperatures the promotor of a small HSP from soy bean is active in all vegetative tissues of Arabidopsis, but not in developing pollen (Crone et al. 2001). The failure of developing pollen to express certain HSP has long been thought to be responsible the high temperature sensitivity of pollen (Frova et al. 1989), and indeed, transgenic overexpression of HSP100, which is not detectable in wild-type pollen of tobacco and cotton under high temperatures, resulted in higher pollen thermotolerance when receiving a short heat stress (46 °C, 3 h or 50 °C, 7 min) during germination and higher bolt and seed production in greenhouse and field trials (Burke and Chen 2015). Recent microarray studies seem to deliver contrasting results, though detecting transcripts of small HSPs, HSP70 and HSP80 and related DNAJ proteins in pollen under short heat stress (Bita et al. 2011; Endo et al. 2009; Zhang et al. 2014). However, these studies used whole anthers or panicles, contributing a significant amount of vegetative tissues that might mask the unique response of developing pollen. Apart from the common HSPs, other putative chaperones have been detected in developing pollen under heat stress (Cooper et al. 1984; Hopf et al. 1992), as well as proteins with chaperone-related functions. Recent studies have shown that members of the BAG family, involved in recruiting HSPs to client proteins, are expressed in developing tomato pollen under heat stress and might be under control of HSFA2 in vegetative tissues in tomato (Fragkostefanakis et al. 2015; Frank et al. 2009; Giorno et al. 2010). Studies in Arabidopsis have shown that these genes are involved in thermotolerance and overexpression resulted in higher tolerance to a variety of other abiotic stresses, too (Doukhanina et al. 2006).

Thus, in agreement with the findings on HSF expression, pollen seems to be able to mount a classical heat stress response regarding the activation of many HSPs, but it is different from that in vegetative tissue.

### Reactive oxygen species

Mitochondria, chloroplasts and peroxisomes are of great importance for energy-related metabolism in plant cells. However, along with the ordinary reactions, these cellular compartments also produce reactive oxygen species (ROS) that are cytotoxic and are detoxified by a specialized cellular machinery (Sharma et al. 2012). While ROS production and scavenging are well balanced under normal conditions, preventing damages to cellular components, abiotic stresses are well known to greatly increase ROS production and disturb this balance (Bhattacharjee 2013; Foyer and Noctor 2005; Vacca et al. 2004). In vegetative tissues of different plant species, ROS scavenging enzymes and antioxidants are known to be highly induced under high temperatures and contribute to plant thermotolerance (Chao et al. 2009; Mittal et al. 2012; Sairam et al. 2000). Increasing antioxidant activity has been shown to increase vegetative thermotolerance in different plant species (Almeselmani et al. 2006; Chen et al. 2013; Rui et al. 1990; Sairam et al. 2000; Sengupta et al. 1993; Wu et al. 2012) and might also be of importance for developing pollen. Pollen and tapetum cells are known to accumulate great numbers of mitochondria, twenty times more than in vegetative tissue, and show fast respiration during development and pollen tube growth (Lee and Warmke 1979; Selinski and Scheibe 2014). Under high temperatures, this great amount of mitochondria might lead to a dramatic increase in ROS, stressing the capacities of the ROS scavenging machinery. The only study into this, performed in wheat, indeed shows that pollen hydrogen peroxide level increases dramatically upon a short heat treatment (42 °C, 2 h), together with antioxidant capacity (Kumar et al. 2014). A proteomic as well as transcriptomic studies have shown that an ascorbate peroxidase is upregulated in developing tomato and wheat pollen in response to a short heat treatment (Chaturvedi et al. 2015; Frank et al. 2009; Kumar et al. 2014). Also in rice, a number of ROS-related genes were shown to be heat responsive (Zhang et al. 2014), although the use of whole panicles prevents drawing conclusions on developing pollen, specifically. When subjected to high temperatures, plants also accumulate antioxidant substances, like flavonoids, that can scavenge and detoxify ROS (Wahid et al. 2007). Accumulation of these antioxidants during pollen development is essential for pollen germination and pollen tube growth (Coberly and Rausher 2003; Derksen et al. 1999; Schijlen et al. 2007). In response to a short heat stress, pollen accumulates even higher levels of ascorbate and phenolic compounds, like flavonoids, that might imply a role in the high temperature response (Kumar et al. 2014).

### Hormones

Various plant hormones have been linked to heat stress signalling and pollen heat acclimation (Bokszczanin et al. 2013; Mittler et al. 2012). Transcripts related to ethylene signalling are higher expressed after a short heat stress in developing tomato pollen (Frank et al. 2009). Supporting a role of ethylene in acclimation of tomato pollen to heat, pollen of an ethylene insensitive tomato mutant was shown to be more sensitive chronic mild heat stress (32/26 °C, day/night), which was associated with reduced accumulation of sucrose in the mature stage (Firon et al. 2012). Additionally, chemical induction of ethylene production prior to a short heat stress (50 °C, 2 h) treatment improved pollen thermotolerance, while application of an ethylene inhibitor reduced it (Firon et al. 2012). Auxin synthesis in anthers of Arabidopsis and barley is reduced upon high temperatures, in contrast to the response in vegetative tissue. This reduction does not seem to be an acclimation response for pollen per se, though, but rather a defect, as exogenous application of auxin improved pollen thermotolerance to continuous mild heat stress in barley (30 °C/25 °C, day/night) and Arabidopsis (31–33 °C) (Higashitani 2013; Sakata et al. 2010). Similarly, a microarray study in rice found that among tapetum-specific genes that were downregulated under continuous mild heat stress (39 °C/30 °C, day/night) several were related to GA signalling (Endo et al. 2009) and a wheat GA hyposensitive mutant was shown to be hypersensitive to long-term high temperature treatments regarding seed set (Alghabari et al. 2014), while a higher GA content correlated with a higher pollen viability under continuous mild heat stress in rice (Tang et al. 2008). Reduced GA signalling may thus be considered a defect, too. Notably, it could be related to developmental defects observed under heat, as a GA insensitive mutant was shown to have abnormal tapetal development, showing delayed or inhibited programmed cell death and pollen developmental arrest at the microspore stage (Aya et al. 2009; Tsuji et al. 2006). Abscisic acid (ABA) is another plant hormone involved in vegetative thermotolerance (Baron et al. 2012; Larkindale and Huang 2005). However, ABA accumulation seems to negatively affect pollen development (Ji et al. 2011; Parish et al. 2013). In rice florets, exposed to reoccurring heat stress for five days, ABA concentrations were higher than under control conditions, but ABA concentrations have not been measured in pollen itself (Tang et al. 2008). Thus, it seems that ABA accumulation could be a consequence of heat stress with adverse effects on pollen. However, evidence is still scarce and further studies that specifically analyse ABA content in anther tissues are needed to shed light on its role in pollen heat stress effects and response.

### Sugar metabolism

The effect of heat stress on pollen characteristics is associated with changes in carbohydrate metabolism and content in the developing anthers, but there are species-specific effects. In reproductive organs of rice, sugar transporters have been shown to be more active under continuous mild heat, resulting in higher starch levels of mature pollen (Chung et al. 2014). Also in bell pepper, mild heat stress was reported to result in higher starch levels in maturing pollen (Aloni et al. 2001). By contrast, in tomato, continuous mild heat stress was found to cause a reduction in cell wall-bound acid invertase activity in whole anthers of flower buds five days before anthesis, which correlated with reduced starch accumulation two days later (Pressman et al. 2006). Acid invertase catalyses hydrolysis of sucrose, which normally accumulates at the final stage of pollen development. The same reaction may be performed by sucrose synthase, and activity of this enzyme, too, has been reported to be negatively affected by high temperature (> 40/25 °C; day/night) in anthers (Kaur et al. 2015). After sucrose is hydrolysed by acid invertase or sucrose synthase, glucose and fructose must be further metabolized. Fructokinase activity, the first step in metabolizing fructose, in bell pepper pollen was also reduced under high temperatures, while hexokinase activity was low and did not show any changes in response to temperature (Karni and Aloni 2002). Interestingly, under higher atmospheric levels of CO_2_, both enzymes showed increased activity and pollen germination potential under high temperatures was improved (Aloni et al. 2001; Karni and Aloni 2002). Following the phosphorylation, hexoses are further metabolized in glycolysis. Genes related to glycolysis are expressed in tapetal cells and developing pollen, and knockout of these genes results in male sterility, accompanied by defects in tapetal development (Munoz-Bertomeu et al. 2010). Whether changes in sugar and starch levels in developing pollen are an adverse consequence of high temperature (via effects on gene expression or enzyme structure/activity) or the result of active adjustments of the primary metabolism as part of the pollen heat response is currently unclear.

## Acquired thermotolerance of pollen development

While plants possess the ability to withstand a sudden heat shock, they have been shown to be more tolerant to a gradual increase in temperature over time that allows them to acclimate. Similarly, plants are able to survive an otherwise lethal heat stress when preceded by a sub-lethal high temperature treatment. This phenomenon is known as acquired thermotolerance (ATT) and has been studied extensively in vegetative tissues. Various genes and signalling pathways have been described that are necessary for ATT; among these are HSFs and HSPs, different plant hormones, ROS and other signals like miRNA398 (Bokszczanin et al. 2013; Guan et al. 2013; Larkindale and Vierling 2008; Scharf et al. 2012). It is thought that upon a sub-lethal high temperature treatment a set of stress signalling and defensive proteins are produced by the plant and remain present in the cell for some time beyond the initial stress period. Then, upon a second heat stress, protective proteins are already available, while the pre-formed signalling components enable the plant to induce transcription of heat-responsive genes faster and at higher levels. As an alternative mechanism, the faster gene expression response has been suggested to depend on histone H3K4 hyper-methylation (Lamke et al. 2015). Among the HSFs and HSPs, some play a very prominent role in ATT; especially, HSFA2 seems to be a major regulator of ATT in Arabidopsis and HSP100 one of the major working horses as knockouts of each of these genes will result in greatly reduced ATT (Hong and Vierling 2000; Schramm et al. 2006; Yang et al. 2006). In vegetative tissues, ethylene signalling has also been related to ATT (Larkindale and Vierling 2008).

While the effect of ATT is described to last for hours up to a few days, there are a few examples that imply that plants might be able to acquire thermotolerance also over longer periods (Charng et al. 2007). In wheat, plants that received two high temperature treatments at earlier developmental stages were performing better when subjected to high temperatures several weeks later, after anthesis (Wang et al. 2011, 2012). In these studies, the authors measured photosynthetic activity of the flag leaf, starch mobilization from the stem and accumulation in the grains. All of them were less affected by high temperatures when plants received acclimation treatments before anthesis.

There is some evidence to suggest that a similar type of ATT also occurs in pollen. Late stages of tomato pollen are able to acquire thermotolerance against a short high temperature stress, and this seems to be dependent on ethylene signalling (Firon et al. 2012). Application of an ethylene inhibitor affected the ability of pollen to acquire thermotolerance and chemical induction of ethylene, prior to a high temperature treatment, increased pollen thermotolerance (Firon et al. 2012). Also, the offspring of Arabidopsis plants grown under continuous mild heat stress for two subsequent generations and one generation at control conditions produced significantly more seeds under continuous mild heat than plants grown under control conditions for three generations prior to the stress (Whittle et al. 2009). Unfortunately, pollen quality was not tested in this study, so it remains unclear whether the increased seed set under high temperatures correlated with increased pollen thermotolerance.

## Conclusions and future perspectives

**Fig. 1:**
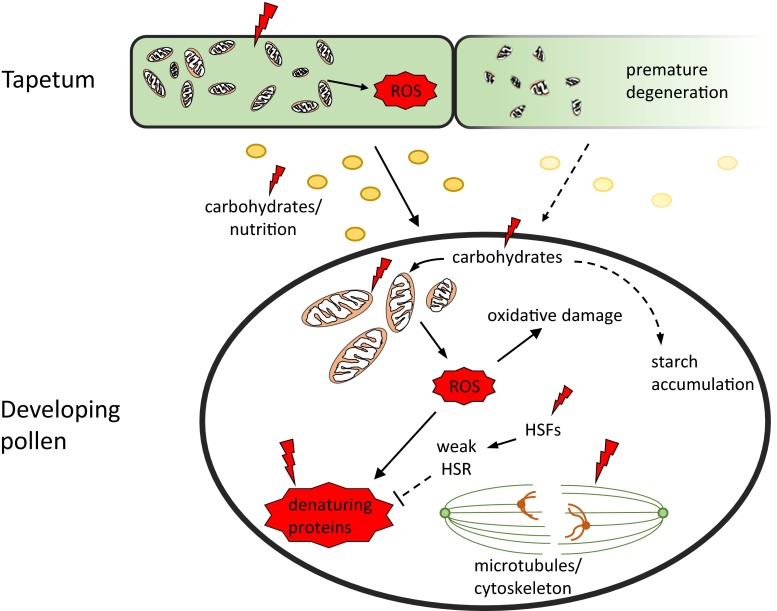
Possible defects related to pollen failure under heat. Developing pollen and the surrounding tapetal cells show a high sensitivity to heat stress (*lightning symbol*) that often results in premature degeneration of tapetal cells and aberrant developmental or programmed cell death of developing pollen. While the cause of this sensitivity remains unknown, we suggest several physiological characteristics of developing pollen that might be related to pollen failure under heat. Firstly, developing pollen and tapetal cells contain high numbers of mitochondria. Therefore, increased respiration as adaptation to heat might result in the production of a disproportional amount of reactive oxygen species (ROS) that cannot be sufficiently detoxified by the protective cellular mechanisms, causing damages to different cellular components. Secondly, premature tapetum degeneration or effects on specific metabolic enzymes under heat stress might result in reduced delivery of carbohydrates and other compounds necessary for normal pollen development. Together, the reduced availability of carbohydrates and the increased respiration with a high number of mitochondria might lead to the depletion of energy reserves and defects during subsequent development. Thirdly, heat results in the unfolding of proteins. This effect is normally mitigated by the classical chaperone heat stress response (HSR). In pollen, heat activates HSFs, important signalling components of the HSR; however, the cell fails to mount a full HSR comparable to vegetative tissues, which is then insufficient to protect and refold proteins. Finally, microtubules and the cytoskeleton are known to be sensitive to ROS and heat stress. During the meiotic cell division, heat affects the orientation of the spindle apparatus leading aberrant chromosome behaviour and subsequent failure of pollen development

While the first detailed studies of pollen development under high temperature date back half a century (Iwahori 1965), the reason for the hypersensitivity of pollen to heat relative to vegetative tissue still remains elusive. We have provided an overview of effects heat on pollen and highlighted differences between the heat stress response of pollen and vegetative tissues. From this, it seems that various factors could contribute to high temperature sensitivity (Fig. 1). One possible explanation could be that pollen is not able to mount a proper HSR. HSFs and HSPs are essential for the heat stress response, and perturbation of this stress defence pathway greatly impairs thermotolerance (Mishra et al. 2002; Schramm et al. 2006). The major HSFs and most HSPs seem to be normally heat responsive in pollen, but several typical HSPs (HSP100 and certain small HSPs) accumulate less in developing pollen than in vegetative tissue under high temperature. Thus, this may hamper the full protection against unfolding of proteins. Support comes from the finding that transgenic expression of Arabidopsis HSP100 in cotton and tobacco improved pollen thermotolerance (Burke and Chen 2015). So, why would a microspore have a weak HSR? It could be speculated that a cell that needs to go through a comp series of developmental steps in a very defined/short period time has limited opportunity to respond to environmental influences. In other words, a strong heat response could be expected to severely affect developmental progression of a microspore, which it might not be able to recover from. Developmental priming has been proposed to compensate for a weak HSR, but it is experimentally difficult to separate heat-independent and heat-dependent protective gene expression, which would be required for testing this hypothesis. Alternatively, specific physiological characteristics of pollen and tapetum might be related to temperature sensitivity. Pollen and tapetum seem to have an unusually strong demand for energy, as indicated by the high number of mitochondria in these cells. Differences in starch and sugar accumulation are usually observed under mild continuous heat conditions. Depletion in energy reserves might thus affect tapetum and pollen more than other cells. Several authors have suggested that problems with sugar metabolism constitute the primary heat defect causing pollen failure, but proper experimental testing is urgently needed to clarify whether this is the case, or whether it is merely the consequence of preceding developmental deviations. From the available literature, it seems that at least pollen induces ROS scavengers upon high temperatures and accumulates compounds that act as antioxidants. However, given the great number of mitochondria in pollen and tapetum, it is tempting to speculate that upon heat, these produce a disproportionate amount of ROS that cannot be counteracted by the detoxifying machinery. Measuring ROS at cell level has proved difficult, explaining the gap in knowledge regarding pollen and tapetum. There might be an opportunity to use genetically encoded biosensors to this end, which may even be evaluated in histological sections (Fujikawa et al. 2016; Meyer et al. 2007). Interestingly, high temperature defects in developing pollen and tapetum share some similarities with plants showing cytoplasmic male sterility (CMS), a phenomenon not completely understood yet, but thought to be linked to mitochondrial function and ROS activity (Hu et al. 2014). About half of the described CMS phenotypes are sensitive to temperatures, and all of them are limited to the development of the male gametophyte. Like under high temperature, aberrations in tapetum development, such as hypertrophy or delayed and inhibited PCD, are observed in CMS plants (Holford et al. 1991; Liu et al. 2009; Schnable and Wise 1998; Smith et al. 2002). This phenotype also closely resembles that of signalling mutants in the GA pathway, which might be involved in pollen thermotolerance (Chhun et al. 2007; Jacobsen and Olszewski 1991; Koornneef and van der Veen 1980; Tang et al. 2008). Whether these similarities are a coincidence or if there is a mechanistic relation in triggering pollen defects remains to be seen.

Taken together, both the development of pollen and tapetum and the high temperature response of developing pollen show some unique characteristics that might explain the high temperature sensitivity of pollen. A contrasting as yet unexplored alternative explanation could be that pollen heat sensitivity is in fact an adaptation by itself. Firstly, because the subsequent processes of embryo and fruit development are adversely affected by high temperature (Bac-Molenaar et al. 2015; Mulholland et al. 2003), preventing investment in reproduction at too high temperatures through regulated reduction in fertility might be beneficial for plant fitness. Support for this hypothesis comes from the specific decrease in auxin levels in anthers upon heat, while in vegetative tissues it increases (Du et al. 2013; Sakata et al. 2010). Insight into the molecular regulation of the two contrasting responses might shed light on this. Secondly, it could be hypothesized that under stress conditions, it could be advantageous to keep the female gametophyte and kill the male gametes. This would promote outcrossing and thus result in higher genetic variability among offspring, which could increase the chance of genetic adaption to adverse conditions. This idea fits with the finding that plants under stress conditions, including heat, show higher homologous recombination frequency (Boyko et al. 2010).

For the future, obtaining knowledge on the genetic basis of (natural) variation in pollen thermotolerance, by applying a forward genetic approach, which has been highly fruitful in many areas of plant research, may be necessary to really get a grip on this aspect of plant biology. Further challenges lie in understanding the similarity in problems caused by and responses to other abiotic stresses, such as drought and high salinity, which also affect male gametophyte development (Storme and Geelen 2013). The common co-occurrence of these stress factors in natural situations argues for studying how they interact.

### **Author contribution statement**

F.M. and I.R. wrote the manuscript. All authors read and approved the manuscript.
